# Proximity labeling uncovers the synaptic proteome under physiological and pathological conditions

**DOI:** 10.3389/fncel.2025.1638627

**Published:** 2025-07-23

**Authors:** Junpei Matsubayashi, Tetsuya Takano

**Affiliations:** ^1^Division of Molecular Systems for Brain Function, Medical Institute of Bioregulation, Kyushu University Institute for Advanced Study, Fukuoka, Japan; ^2^PRESTO, Japan Science and Technology Agency, Saitama, Japan

**Keywords:** synapse, proteomics, BioID, spine formation, cytoskeleton, synaptopathy

## Abstract

Synapses are fundamental units of neurotransmission and play a central role in the formation and function of neural circuits. These dynamic structures exhibit morphological and functional plasticity in response to experience and activity, supporting higher brain functions such as learning, memory, and emotion. Their molecular composition includes diverse membrane-associated and cytoskeletal proteins that mediate intercellular signaling, regulate synaptic plasticity, and maintain structural stability. Disruptions in these protein networks, often referred to as synaptopathies, are closely linked to psychiatric and neurological disorders. Such disruptions commonly manifest as region-specific changes in synapse number, morphology, or signaling efficacy. Although a large number of synaptic proteins have been identified through conventional proteomic approaches, our understanding of synaptic specificity and plasticity remains limited. This is primarily due to insufficient spatial resolution, lack of cell-type specificity, and challenges in applying these methods to intact neural circuits *in vivo*. Recent advances in proximity labeling techniques such as BioID and APEX can spatial proteomics limiting cell compartments and cell-type. BioID also enables proteomic analysis within synaptic compartments under both physiological and pathological conditions *in vivo*. These technologies allow unbiased, high-resolution profiling of protein networks in specific synapse types, synaptic clefts, and glial-neuronal interfaces, thereby providing new insights into the molecular basis of synaptic diversity and function. In this short review, we summarize recent developments in synaptic proteomics enabled by proximity labeling. We also discuss how these approaches have advanced our understanding of synapse-specific molecular architecture and their potential to inform the mechanisms of synapse-related brain disorders, as well as the development of targeted diagnostic and therapeutic strategies.

## Introduction

Synapses are highly specialized subcellular compartments of neurons and represent the fundamental computational units for neurotransmission. Each neuron connects to thousands of others via asymmetric intercellular junctions composed of presynapses and postsynapses, facilitating continuous signal transmission. In the human brain, approximately 150 trillion synapses form intricate neural circuits across various brain regions. These synapses undergo dynamic morphological and functional plasticity throughout life, influenced by environmental stimuli such as sensory experience and behavioral activity besides to the genetic programs. This remarkable plasticity underpins fundamental brain functions such as learning, memory, and emotion by supporting adaptive modifications in synaptic connectivity and strength. Structurally, synapses are composed by synaptic vesicles, the presynaptic active zone, the synaptic cleft, and the postsynaptic density (PSD), each compartment drives the above brain functions in a coordinated manner. Notably, thousands of distinct synaptic proteins orchestrate the brain functions and the structural of synapses, and define the discrete synaptic properties in the different brain regions (O’Rourke et al., 2012; Koopmans et al., 2019; Van Oostrum et al., 2023). Also, synaptic proteins include cytoskeletal proteins, receptors, neurotransmitters, adhesion molecules and scaffold proteins (O’Rourke et al., 2012; Koopmans et al., 2019), and this molecular diversity exemplifies how synaptic components are not merely structural but actively shape signaling integration, synapse specification, and plasticity. This functional complexity is made possible by the spatially confined and molecularly compartmentalized organization of synapses, which enables precise and localized biochemical processing. Thus, each synapse operates as a self-regulating biochemical microdomain capable of adaptive computation within neural circuits. Conversely, the dysfunction of synaptic protein networks leads to impairments in synapse number, morphology, and signal transmission. Accumulating evidence suggests that such synaptic dysfunctions, which are often referred to as synaptopathies, are closely associated with neurodevelopmental and psychiatric disorders as well as with the progression of neurodegenerative diseases (Grant, 2012; Lepeta et al., 2016; Hindley et al., 2023). Notably, these abnormalities often manifest in specific brain regions. Although large-scale efforts have led to the identification of over 2,000 distinct synaptic proteins through conventional proteomic approaches (Bayés et al., 2011; Loh et al., 2016; Koopmans et al., 2019), the molecular mechanisms governing synaptic specificity, diversity, and plasticity remain incompletely understood. This is due, in part, to limitations in spatial resolution, cell-type specificity, and the ability to analyze intact neural circuits *in vivo*.

In recent years, emerging proximity labeling (PL)-based proteomic techniques such as BioID (biotin ligase-based), APEX (ascorbate peroxidase) and HRP (horseradish peroxidase) have made it possible to profile the local proteome of synaptic compartments with high spatial resolution in living tissue (Han et al., 2018; Takano and Soderling, 2021; Ito et al., 2024). These techniques have facilitated the discovery of proteomes associated with specific neuronal populations (Uezu et al., 2016), synaptic clefts (Loh et al., 2016; Takano et al., 2020), and tripartite synapses formed by astrocyte-neuron connections (Takano et al., 2020; Takano and Soderling, 2021). In this short review, we highlight recent advances in spatial synaptic proteomics enabled by proximity labeling (PL) technologies and discuss how these approaches have advanced our understanding of the molecular mechanisms underlying synapse formation, diversity, and function. We further introduce current insights into the pathophysiology of synapse-related neurological disorders uncovered through PL-based studies and outline future directions for the therapeutic application of these technologies. By enabling precise profiling of synapse-specific molecular networks, PL-based proteomic approaches offer novel insights into brain function and hold considerable promise for the development of targeted diagnostic and therapeutic strategies for synapse-associated disorders.

## Proximity labeling approaches for synaptic protein profiling

Traditionally, synaptic proteins have been identified using liquid chromatography-tandem mass spectrometry (LC-MS/MS) analysis of synaptic vesicles and synaptosomes, purified by differential centrifugation, density-gradient centrifugation, immune-purification and affinity-purification (Fernández et al., 2009; Morciano et al., 2009; Grønborg et al., 2010; Boyken et al., 2013; Wilhelm et al., 2014; Dieterich and Kreutz, 2016; Xu et al., 2021; Kaizuka et al., 2024). While these proteomic approaches have proven valuable for detecting synaptic proteins enriched in cultured neurons and brain tissues, they are limited by low spatial resolution and contamination from heterogeneous mixtures derived from multiple synapse types. These limitations hinder the ability to resolve the molecular characteristics of specific cell types, synapse subtypes, synaptic clefts, and tripartite synapses.

**FIGURE 1:**
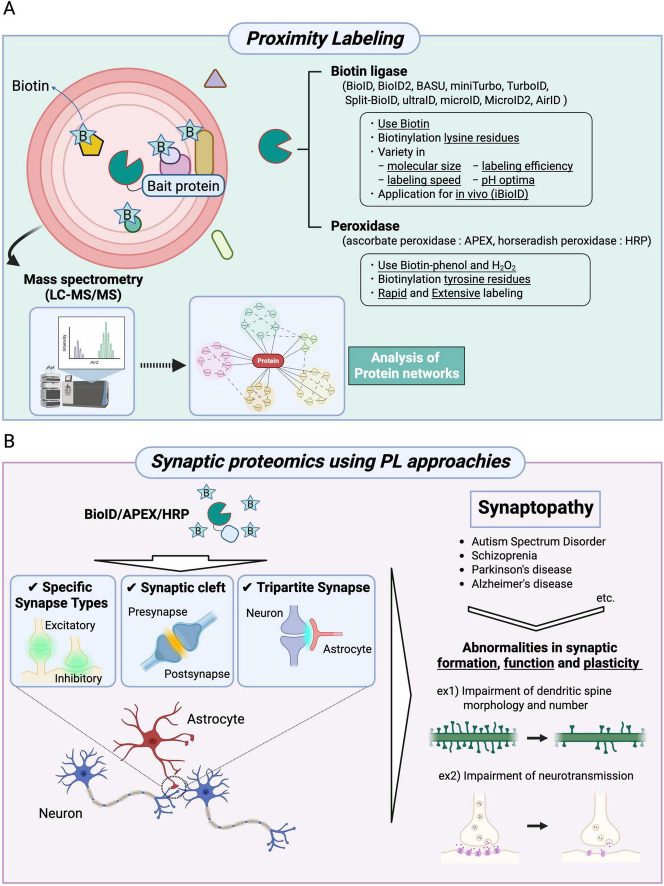
Synaptic proteomics approaches using proximity labeling to uncover physiological and pathological conditions. **(A)** A schematic diagram and applications of BioID, APEX, and HRP are shown. The proteins of interest (bait proteins) are fused with BioID (a biotin ligase), APEX (ascorbate peroxidase), or HRP (horseradish peroxidase) and expressed in cells. BioID biotinylates lysine residues of proteins in proximity to the bait protein, whereas APEX and HRP biotinylate tyrosine residues of nearby proteins. In the BioID approach, various types of biotin ligases can be selected. Moreover, BioID technologies can be applied to *in vivo* studies (iBioID). The biotinylated proteins are identified using mass spectrometry, followed by analyses of molecular localization and function based on the constructed protein networks. **(B)** Proximity labeling methods (BioID, APEX, and HRP) enable high spatial resolution mapping of proteins localized to specific synapse types, the synaptic cleft, and tripartite synapses. These synaptic proteomics approaches have also been applied to the study of synaptopathies, including autism spectrum disorder, schizophrenia, Parkinson’s disease, and Alzheimer’s disease. These neuropsychiatric disorders are characterized by abnormalities in synapse formation, function, and plasticity.

In recent years, PL technologies such as BioID, APEX, and HRP have emerged as powerful biochemical tools for spatially resolved synaptic proteomics (Ito et al., 2024; Table 1). These approaches rely on enzyme-mediated biotinylation of proteins located in the immediate vicinity of a target protein fused to a biotin ligase or peroxidase. Biotinylated proteins are subsequently purified using streptavidin, NeutrAvidin, anti-biotin antibody and Tamavidin 2-REV-coated beads, followed by identification using LC-MS/MS (Figure 1A). BioID, the first biotin ligase-based PL method, uses a mutant *Escherichia coli* biotin ligase (BirA*-R118G) that generates reactive biotin (biotinoyl-5’-AMP) and biotinylates lysine residues of nearby proteins in the presence of biotin (typically within ∼10–20 nm) (Han et al., 2018; Ito et al., 2024; Table 1). Since the original development of BioID, a broad spectrum of proximity-labeling ligases has been engineered to enhance properties such as molecular size, catalytic efficiency, labeling kinetics, and specificity under various physiological and pathological condition. BioID2 is a truncated variant of BioID that retains proximality labeling capability while offering improved efficiency due to its smaller size (Kim et al., 2016). BASU enables more than 1,000 times faster kinetics and more than 30 times increased signal-to-noise ratio over the prior BioID (Ramanathan et al., 2018). TurboID and miniTurbo show much greater efficiency than BioID and BioID2, and biotinylate proteins for 10 min (Branon et al., 2018). Labeling speed of TurboID (∼1 h) is much faster than BioID (∼12–16 h), but TurboID has strong biotinylation activity, which may cause non-specific labeling (Table 1). Split-BioID is splitting BirA into two parts, fusing each fragment with a different protein, and reactivating the BirA enzyme when the complex is formed (De Munter et al., 2017; Schopp et al., 2017; Cho et al., 2020; Takano et al., 2020). The microID, a truncation variant of BioID, is a small-sized biotin ligase and shows efficient Biotinylation at short labeling times (Kubitz et al., 2022). The ultraID is also the directed evolution of microID (Kubitz et al., 2022). MicroID2 is a modified BioID2 and enables lower background labeling than TurboID (Johnson et al., 2022). AirID was engineered by *in silico* design and shows low ability to biotinylate proteins non-specifically (Kido et al., 2020; Table 1). In this way, researchers can select appropriate tools based on specific experimental needs. In contrast, APEX and HRP are peroxidase-based PL methods that catalyze biotinylation of tyrosine residues using reactive radicals generated from biotin-phenol and hydrogen peroxide (H_2_O_2_) (Table 1). HRP is mainly used for biotinylation of extracellular proteins, because HRP requires intramolecular disulfide bonds, but disulfide bond formation is basically difficult inside cells. On the other hand, APEX can use intracellular labeling because it does not require disulfide bonds. These two peroxidase-based PL methods enable rapid (seconds to minutes) and extensive labeling (APEX: 20 nm, HRP: 200–300 nm) than BioID (∼10 nm). However, the important point to note is that these approaches are mainly restricted to *in vitro* or *ex vivo* applications due to the cytotoxicity of H_2_O_2_ (Figure 1A and Table 1).

**TABLE 1:** Summary of proximity labeling technologies.

Proximity labeling	Type	Applications		Molecular weight (kDa)	Labeling				Advantages	Disadvantages
					Residues	Molecules	Time	Radius		
BirA	Biotin ligase	Intracellular extracellular	*In vitro in vivo*	35	Lysine	Biotinoyl-5’-AMP	12–16 h	∼10 nm	Suitable for *in vivo* applications because of non-toxic (*in vivo* BiolD)	• Low biotinylation activity (long labeling time required) • High biotin concentration is required
TurboID	Biotin ligase	Intracellular extracellular	*In vitro in vivo*	35	Lysine	Biotinoyl-5’-AMP	Within 1 h	∼10 nm	Suitable for *in vivo* applications because of non-toxic (*in vivo* BiolD) High biotin labeling potential and quick reaction	• Non-specific biotinylation by high biotin labeling potential
AirID	Biotin ligase	Intracellular extracellular	*In vitro in vivo*	37	Lysine	Biotinoyl-5’-AMP	Within 3 h	10–20 nm	Available low biotin concentration ’Wide range of optimal temperatures (15-45X)	• Middle labeling time required
APEX	Peroxidase	Intracellular extracellular	*In vitro ex vivo*	28	Tyrosine Tryptophan Cysteine Histidine	Radical biotin phenol	Seconds to minutes	20 nm	Fast reaction more than biotin ligase-based PL	• No suitable for apply *in vivo* because of the H_2_O_2_ cytotoxity
HRP	Peroxidase	Extracellular	*In vitro ex vivo*	44	Tyrosine Tryptophan Cysteine Histidine	Radical biotin phenol	Seconds to minutes	200–300 nm	Fast reaction more than biotin ligase-based PL	• No suitable for intracellular labeling • No suitable for apply *in vivo* because of the H_2_O_2_ cytotoxity

Notably, BioID approaches have enabled the mapping of synaptic proteins in the brain (Uezu et al., 2016). This *in vivo* BioID (iBioID) technique, in combination with genetic tools such as adeno-associated virus (AAV) vectors and transgenic mice, permits targeted profiling of synaptic proteomes in specific circuits and cell types (Uezu et al., 2016; Ito et al., 2024). However, unlike *in vitro*, it is necessary to administer biotin continuously for several days. More recently, Cho et al. (2025) introduced a membrane-tethered version of HRP (HRP-TM) that utilizes endogenously generated H_2_O_2_ for cell surface biotinylation *in vitro*, offering potential for application without the need for exogenous H_2_O_2_. In another advancement, Zhang et al. (2025) reported TyroID, a novel tyrosinase-based PL technique, that enables non-toxic labeling of various nucleophilic residues both *in vitro* and *in vivo*, using reactive o-quinone intermediates derived from phenol-based probes such as alkyne-phenol or biotin-phenol (Zhang et al., 2025). These tools have not yet been applied to spatial synaptic proteomics, but they are expected to be novel *in vivo* PL tools that compensate for the poor temporal resolution of iBioID. Collectively, PL technologies are rapidly evolving to support *in vivo* applications and, when combined with virus-based chemogenetic tools such as AAV and transgenic mouse systems, offer a powerful platform for dissecting the molecular mechanisms of synapse formation and function at high spatial resolution.

## Synapse-type-specific proteomics using PL approaches

### Proximity labeling-based profiling of specific synapse types

Chemical synapses, which serve as the primary sites of neurotransmission, are broadly classified into excitatory or inhibitory synapses in the brain. These synapses exhibit distinct morphological features including synaptic vesicle shape, presynaptic density, and active zone size that vary depending on cell type, brain region, and molecular composition (Van Oostrum et al., 2023; Van Oostrum and Schuman, 2025). Conventional methods for synapse-targeted proteomics lack the spatial resolution necessary to distinguish between specific synapse types. In contrast, PL approaches allow for the precise analysis of defined synapse types both *in vitro* and *in vivo*. Using a iBioID strategy, Uezu et al. (2016) identified 121 unique proteins at excitatory synapses and 181 proteins at inhibitory synapses (Uezu et al., 2016; Table 2). Among these synaptic proteins, a previously uncharacterized protein, InSyn1, was found to localize to inhibitory postsynaptic sites. Additionally, they found that InSyn1 regulates miniature excitatory postsynaptic current (mIPSC) by interacting with the dystrophin complex in the hippocampus. Spence et al. (2019) examined the proteome of the developing dendritic filopodia during excitatory synaptogenesis using Wrp (Rac-GAP)-BirA for iBioID labeling. This approach identified 60 synaptic candidate proteins and revealed that CARMIL3, a previously uncharacterized protein, contributes to spine maturation and synapse unsilencing by interacting with WRP and actin capping protein within nascent dendritic spines (Spence et al., 2019; Table 2). Moreover, Falahati et al. (2022) investigated the molecular components of the spine apparatus using synaptopodin-fused BioID2 in the mouse brain (Falahati et al., 2022; Table 2). This approach identified 140 proteins and found that Pdlim7, an actin-binding protein, coassembles with synaptopodin and actin to regulate dendritic spine structure (Falahati et al., 2022). Recently, Rosenthal et al. (2025) explored the development and plasticity of central cholinergic synapses by performing *in vivo* spatial synaptic proteomics (Table 2). Using CRISPR/Cas9, they inserted miniTurboID into Dα1 and Dα6 subunits of nicotinic acetylcholine receptors (nAchRs) in developing and mature Drosophila brains. Proteomic analysis identified 81 core proteins associated with nAchR function and revealed that the Rho-GTPase regulator Still life (Sif) acts as a key structural organizer of cholinergic synapses through interactions with postsynaptic density components (Rosenthal et al., 2025). In addition to chemical synapses, a recent work has extended PL technologies to electrical synapses in retinal neurons. Tetenborg et al. (2025) employed TurboID-fused Connexin 36 (Cx36), a major neuronal gap junction protein, to profile electrical synapses in zebrafish and mouse retinas. Using two different TurboID strategies in zebrafish and mice, they identified more than 50 novel synaptic proteins and demonstrated that signal-induced proliferation-associated 1-like 3 (SIPA1L3) regulates synaptic density by interacting with Cx36, thereby contributing to electrical synapse formation (Table 2). Together, these studies demonstrate that PL-based approaches enable high-resolution and synapse-type-specific proteomic profiling, including chemical synapses, such as excitatory and inhibitory synapses and electrical synapses, thus providing a powerful platform for dissecting the molecular architecture of diverse synapse types in the brain (Figure 1B).

**TABLE 2:** Summary of synaptic proteomics using proximity labeling to uncover molecular orchestrations in the brain.

Focus	Target	Proximity labeling	Gene transduction method	Brain region	Cell type	Subcellular compartments	The number of identified proteins	Focus protein	Effects on synapses	References
Synapse and functions	Specific synapse type	PSD95-BirA (postsynaptic density 95 protein-fused BirA)	AAV vector (AAV: adeno-assoociated virus)	Mouse Hippocampus (*in vitro*)	Neurons (Excitatory)	Excitatory post-synapse	121	–	–	Uezu et al., 2016
		Gephyrin-BirA (gephyrin-fused BirA)	AAV vector	Mouse Hippocampus (*in vitro*)	Neurons (inhbitory)	Inhibitory post-synapse	181	(Previously uncharacterized protein)	Abnormal synaptic inhibition	Uezu et al., 2016
		Wrp-BirA (Synaptic cytosleletal regulator proteins SrGAP3-fused BirA)	AAV vector	Mouse Hippocampus (*in vitro*)	Neurons	Nascent dendritic spine	60	CARMIL3 (actin regulator protein)	Dendritic protrusion density X Dendritic spine maturation X Excitatory synaptic alteration	Spence et al., 2019
		BioID2-synaptopodin (BioID2-fusing synaptopodin, a spine apparatus-specific protein)	AAV vector	Mouse Hippocampus	Neurons	Dendritic spine	140	Pdlim7 (actin-binding protein)	–	Falahati et al., 2022
		Da1-miniTurboID Da6-miniTurboID (Dal or Da6, drosophila nAchR subunits-fused miniTurbolD)	CRISPR/Cas9 genome editing	Dorosofila Brain	Neurons	nAchRs	81	Sif (Rho-GTPase regulator)	Synaptic density X (loss of function)	Rosenthal et al., 2025
		Cx36-TurboID (conn-fused TurboID)	AAV vector	Mouse Retina	Neurons	Electrical synapse	50	SIPA1L3 (scaffold protein)	Synaptic density X (knock out)	Tetenborg et al. (2025)
	Synaptic cleft	HRP-Lrrtm1 HRP-Lrrtm2 (HRP-fusing Lrrtm1 or Lrrtm2, glutamatergic excitatory synaptic cleft-resident proteins)	Lentivirus vector	Rat Cortex (*in vitro*)	Neurons	Glutamatergic excitatory Synaptic cleft	199	–	–	Loh et al., 2016
		HRP-Slitrk3 HRP-Nlgn2 (HRP-fusing Slitrk3 or Nlgn2, GABAergic inhibitory synaptic cleft resident proteins)	Lentivirus vector	Rat Cortex (*in vitro*)	Neurons	GABAergic excitatory Synaptic cleft	42	Mdga2 (postsynaptic membrane protein)	Inhibitory synapse density f (overexpression)	Loh et al., 2016
		HRP-Lrrtm1 HRP-Lrrtm2	Lentivirus vector	Rat Cortex (*in vitro*)	Neurons	Glutamatergic excitatory Synaptic cleft (activity-driven exocytosis of endogenous proteins)	–	–	–	Pascual-Caro and De Juan-Sanz, 2024
		SynCAM1-HRP (SynCAM1, excitatory synaptic cell adhesion protein-fused HRP)	AAV vector	Rat Cortex (*in vitro*)	Neurons	Excitatory Synaptic cleft	39	R-PTP-Z (Receptor-type tyrosine-protein phosphatase zeta)	**–**	Cijsouw et al., 2018
		TurboID-surface (GPI anchor-fused TurboID to selectively label membrane-associated proteins)	AAV vector	Mouse Cortex	Astrocytes	Plasma membrane	178	–	–	Takano et al., 2020
		Split-TurboID (splitted TurboID enzyme into N-terminal and C-terminal fragments, reconstitution only at the cellular contact site)	AAV vector	Mouse Cortex	Neurons (N TurboID) Astrocytes (C TurboID)	Tripartite synaptic clefts	173	NRCAM (neuronal cell adhesion molecule, expressed in Astrocyte)	Inhibitory synapse density X (knock down) mIPSC X (knock down)	Takano et al., 2020
		TurboID-NCAN-ELS (TurboID-fusing neurocan C-terminal ELS domain)	AAV vector	Mouse Cortex	Astrocytes	Tripartite synaptic clefts	166	NCAN C-terminal fragment	SST (inhibitory synapse formation X (mutant mouse)	Irala et al., 2024
Synapse-related disorder	Parkinson’s disease	Densin-180-BioID2 (Densin-180, a postsynaptic scaffold at glutamatergic synapses-fused BioID2)	DNA trancfection	–	HEK293T	cytoplasm	–	PP1a (protein phosphatase 1)	–	Willim et al., 2024
	Autism spectrum disease (ASD)	TurboID knock-in 14 ASD proteins (using HiUGE-iBioID) (Anks1b, Syngap1, Shank2, Shank3, Nckap1, Nbea, Ctnnb1, Lrrc4c, Iqsec2, Arhgef9, Ank3, Scn2a, Scn8a, and Hnrnpu)	AAV vector	Whole Brain	Neurons	Synapses Axon initial segment Nucleus	1,252	SynGAP1 (ras GTPase-activating protein 1) Scn2a (sodium channel protein type 2 subunit alpha)	Neural activity X (SynGAP1, knock down) Repetitive behaviors (Scn2a, mutant mouse) Abnormal communication (Scn2a, mutant mouse) Neural activity X (Scn2a, mutant mouse)	Gao et al., 2024
	Parkinson’s disease	Ezrin-BioID (Ezrin, a crucial linker between the cell membrane and the actin cytoskeleton-fused BioID)	AAV vector	Whole Brain	Astrocytes	cytoplasm	344	Atg7 (autophagy regulator)	Astrocytic territory volume X (knock down)	Wang et al., 2023

### Proximity labeling-based profiling of synaptic cleft and tripartite synapse

The synaptic cleft is a highly specialized extracellular compartment, approximately 20 nm in width, formed between the presynaptic and postsynaptic membranes. It plays a critical role in neurotransmission by mediating cell-cell communication through a dense array of adhesion molecules, receptors, and neurotransmitters. Accurate characterization of the protein components within synaptic clefts is therefore essential for understanding the molecular basis of synaptic function and plasticity. Loh et al. (2016) developed a peroxidase-based synaptic cleft proteomes in cultured neurons by expressing HRP-tagged versions of cleft-resident adhesion molecules, including Lrrtm1, Lrrtm2, Slitrk3 and Nlgn2, which are selectively enriched in excitatory or inhibitory synapse (Table 2). This proteome analysis identified 199 glutamatergic and 42 GABAergic proteins, including Mdga2, a previously uncharacterized protein localized at inhibitory synaptic clefts. Further, functional analysis revealed that Mdga2 regulates recruitment of presynaptic terminals to inhibitory postsynapses through interaction with Neureglin-2 (Loh et al., 2016). Also, Pascual-Caro and De Juan-Sanz (2024) introduced an HRP-based approach to label neural activity-driven trafficking of endogenous synaptic proteins by fusing HRP to Lrrtm1 and Lrrtm2 (Pascual-Caro and De Juan-Sanz, 2024; Table 2). Similarly, Cijsouw et al. (2018) used HRP-tagged SynCAM1, an excitatory synaptic adhesion molecule, to identify receptor-type tyrosine-protein phosphatase zeta (R-PTP-ζ) as a novel candidate synaptic cleft protein in cortical neurons (Table 2). These studies demonstrate that PL-based strategies can selectively label proteins localized within the synaptic cleft, thereby minimizing contamination from intracellular components and enabling precise mapping of extracellular synaptic interfaces (Figure 1B).

In the brain, the astrocyte, which is the most abundant glial cell in the brain, interact with neurons at specialized contact sites to modulate synaptic function and circuit remodeling. These astrocyte-synapse junctions, referred to as tripartite synapses, play crucial roles in the regulation of neurotransmission, synaptic plasticity, and brain homeostasis (Takano and Soderling, 2021; Farizatto and Baldwin, 2023; Raghunathan and Eroglu, 2025). However, conventional proteomic approaches often struggle to resolve such contact-dependent molecular interactions due to the high degree of cellular heterogeneity and the complex intermingling of neural structures within the brain. To overcome these challenges, Takano et al. (2020) developed two innovative proximity labeling (PL)-based techniques: TurboID-surface and Split-TurboID (Takano et al., 2020; Takano and Soderling, 2021). TurboID-surface utilizes a glycosylphosphatidylinositol (GPI) anchor-fused TurboID to selectively label membrane-associated proteins. Split-TurboID separates the TurboID enzyme into N-terminal and C-terminal fragments, which are individually expressed in distinct cell types and become functionally reconstituted only at the cellular contact site (Takano et al., 2020). By integrating these tools with cell type-specific AAV, they performed spatial proteomic profiling of tripartite synapses in the mouse brain, identifying 118 proteins enriched at astrocyte-neuron junctions. Interestingly, neuronal cell adhesion molecule (NRCAM) was found to be strongly localized at perisynaptic astrocytic processes and was shown to facilitate the formation and function of inhibitory postsynapses. This effect is mediated through the recruitment of gephyrin via homophilic interactions between neuronal and astrocytic NRCAM (Takano et al., 2020; Table 2). In addition to this approach, Irala et al. (2024) investigated astrocyte-derived secreted factors that influence the development of inhibitory synapses (Irala et al., 2024). They engineered a secreted form of TurboID fused to the neurocan (NCAN)-ELS domain, which contains synaptogenic protein interaction motifs, and introduced it into astrocytes using an AAV vector. This approach revealed that the C-terminal fragment of astrocyte-secreted NCAN plays a key role in regulating the formation and functional maturation of somatostatin-positive inhibitory synapses in the developing mouse cortex (Irala et al., 2024; Table 2). Together, these studies demonstrate the high versatility and spatial precision of PL-based approaches such as TurboID-surface and Split-TurboID for analyzing protein networks at specialized subcellular and intercellular sites. When coupled with viral gene delivery and cell type-specific expression systems, these methods provide a powerful experimental platform for elucidating the molecular architecture of complex cellular interactions, including those at synaptic clefts and tripartite synapses, under both physiological and pathological conditions (Figure 1B).

### Synaptopathy-focused proteomics using PL approaches

A previous great number of studies demonstrate that synaptopathies, defined as abnormalities in synaptic formation, functions, and plasticity, are common pathological features of neurodevelopmental disorders such as autism spectrum disorder (ASD), psychiatric disorders like schizophrenia and neurodegenerative diseases such as Parkinson’s disease (PD) and Alzheimer’s disease (Grant, 2012; Lepeta et al., 2016; Hindley et al., 2023; Figure 1B). Therefore, elucidating the molecular basis of synaptopathies represents a crucial step toward a comprehensive understanding of their pathophysiology and the development of targeted therapeutic strategies. In recent years, spatial synaptic proteomics using PL technologies has emerged as a powerful and versatile approach to uncover the molecular architecture and dynamic regulation of synapses under both physiological and pathological conditions. These technologies enable high-resolution mapping of protein interactions and local proteomes in defined synaptic compartments and cell types, thereby offering novel insights into the mechanisms underlying synapse-related neurological disorders (Figure 1B).

Previous studies have shown that Densin-180, a PSD protein encoded by *LRRC7*, is highly expressed at excitatory synapses. Densin-180 deficient mice show impaired long-term depression and memory formation and aggressive behavior (Strack et al., 2000; Carlisle et al., 2011; Chong et al., 2019). Recently, Willim et al. (2024) reported that human variants in *LRRC7* are associated with neurodevelopmental disorders including intellectual disability, autism, aggression and abnormal eating behaviors (Willim et al., 2024). In this study, PL screening using BioID2-fused Densin-180 identified protein phosphatase (PP1α), another PSD component, as a strong interactor with the leucine rich repeat (LRR) domain of Densin-180 in HEK293T cells. Functional analysis revealed that disease-associated LRR domain variants disrupt binding to PP1α. These findings suggest that Densin-180 scaffolds PP1α to its postsynaptic substrates, and that disruption of this interaction impairs synaptic signaling, contributing to the observed behavioral and cognitive abnormalities (Willim et al., 2024; Table 2). Gao et al. (2024) developed a high-throughput PL screening platform that combines iBioID using TurboID with Homology independent Universal Genome Engineering (HiUGE) approach, which is a CRISPR/Cas9-based genome editing system to investigate 14 risk genes of ASD. Using these approaches, they identified 1,252 interacting proteins. Among these, they focused on Syngap1, a synaptopathy-related protein, and Scn2a, a channelopathy-related protein. Notably, PL and immunoblot analysis showed that an autism-associated mutation of Syngap1 disrupts its interaction with Anks1b, and this interaction is essential for the formation of neural activity in the crucial period of synaptogenesis (Gao et al., 2024; Table 2). Additionally, a patient-derived mutation of Scn2a exhibited repetitive behaviors and deficits in social communication. Proteomic analysis showed that these mutants displayed downregulation of Scn1b and Fgf12, both key modulators of Scn2a function. Importantly, restoring the expression of these proteins rescued the abnormal electrophysiological phenotypes, highlighting their therapeutic potential (Gao et al., 2024; Table 2). Wang et al. (2023) investigated the effect of PD-associated G2019S mutation in the leucine-rich repeat kinase 2 (LRRK2) on the synaptic functions (Wang et al., 2023). Using BioID-fused Ezrin, a protein highly expressed in astrocytes, they identified autophagy-related 7 (Atg7) as an binding partner. Further analysis using LRRK2 G2019S*^ki/ki^* mice revealed that phosphorylation of Ezrin disrupts its interaction with Atg7, leading to dysregulated astrocyte morphology and impaired synaptic connectivity. These findings suggest that astrocyte dysfunction caused by LRRK2 mutation contributes to synaptic pathophysiology in PD (Wang et al., 2023; Table 2). Collectively, these studies demonstrate that PL-based proteomics can illuminate the molecular pathways underlying synaptopathies (Figure 1B). By mapping protein interactions in disease-relevant synaptic contexts, PL approaches offer new avenues for therapeutic development.

## Concluding remarks and outlook

Proximity labeling approaches have significantly advanced the field of synaptic proteomics by enabling molecular profiling of subcellular compartments with high spatial precision. Applications of BioID, APEX, and HRP have facilitated the identification of proteomes in various synaptic environments, including excitatory and inhibitory synapses (Uezu et al., 2016), cholinergic (Rosenthal et al., 2025) and electrical synapses (Tetenborg et al., 2025), as well as glial interfaces such as tripartite synapses (Takano et al., 2020). These studies have expanded the catalog of synaptic proteins and provided valuable insights into how synapses are assembled, maintained, and modified in both healthy and diseased brains (Figure 1 and Table 2).

Nevertheless, several technical and conceptual challenges must be addressed to fully harness the potential of PL-based approaches. One major limitation is the low temporal resolution of current labeling systems. BioID-based methods typically require several days of biotin supplementation to achieve effective labeling *in vivo*, which makes it difficult to capture rapid or transient protein interactions that occur in response to neuronal activity or environmental changes. In contrast, APEX and HRP allow for much faster labeling (≤1 min) but depend on hydrogen peroxide, which is cytotoxic and unsuitable for applications in intact brain tissue. Although new methods such as HRP-TM, which utilizes endogenous hydrogen peroxide (Cho et al., 2025), and TyroID, which employs non-toxic o-quinone chemistry (Zhang et al., 2025), offer promising alternatives, their specificity and applicability in complex brain tissue remain to be fully validated. Further *in vivo* studies are expected to assess their performance under physiological and pathological conditions, particularly in identifying activity-dependent or circuit-specific proteomic changes within intact neural networks. Another important issue is the limited spatial resolution of current labeling methods. In the brain, synapses are highly compact structures where proteins from presynaptic neurons, postsynaptic neurons, and surrounding glial cells are densely intermingled. Although, TurboID can label proteins within a radius of approximately 10 nm, its biotynilation activity is so potent compared to BioID (Branon et al., 2018). Therefore, it may biotinylate not only the intended molecular targets but also nearby proteins from adjacent compartments. This overlap makes it difficult to determine exactly where the labeled proteins are localized within the synapse. To overcome this limitation, recent methods such as Split-HRP (Martell et al., 2016) and Split-TurboID (Cho et al., 2020; Takano et al., 2020) have been developed. This technique divides the labeling enzyme into two inactive fragments that only reconstitute and become active when two different cell types are in direct contact. Using this strategy, we successfully identified molecules such as NRCAM that localize specifically to astrocyte-neuron interfaces and play a critical role in organizing inhibitory synapses (Takano et al., 2020). These findings highlight how cell-contact-dependent labeling can improve spatial precision and uncover new mechanisms of synaptic regulation.

Quantitative interpretation of PL data also remains a challenge. Currently, there is no widely accepted standard for normalization, statistical comparison, or integration of PL proteomes across different developmental stages or disease models. Combining PL-based proteomic data with complementary approaches such as single-cell transcriptomics (Yao et al., 2023; Zhang et al., 2023), spatial transcriptomics (Lein et al., 2017; Yuan et al., 2025), and high-resolution imaging (Newman et al., 2022; Unterauer et al., 2024) will likely be necessary to interpret the data in a biologically meaningful context. For instance, a recently published single-cell mass cytometry-based atlas of the developing mouse brain provides a valuable resource for anchoring synaptic proteomic data within a broader cellular and developmental framework (Van Deusen et al., 2025). The combined analysis of this advanced technology and PL approaches could also offer a key resource for future novel “single-synapse proteome” research field. Additionally, PL methods have provided important insights into the molecular mechanisms of neurological and psychiatric disorders (Table 2). Recent studies using disease models or patient-derived mutations have shown how alterations in protein–protein interactions can impair synaptic signaling and lead to behavioral and cognitive deficits. For example, disrupted interactions between Densin-180 and PP1α (Willim et al., 2024) as well as changes in the Scn2a-associated proteome (Gao et al., 2024) have been linked to neurodevelopmental disorders. These findings highlight the utility of PL techniques for mechanistic investigations and therapeutic target identification besides for mapping molecular discovery (Figure 1B). In summary, proximity labeling-based synaptic proteomics represents a powerful platform for investigating the molecular logic of synapse formation, function, and dysfunction. Also, synaptic proteomics has greatly advanced our understanding of molecular diversity within synapses, and revealing a number of unknown molecular mechanisms involved in this diversity may provide cues to decoding the intricate brain functions induced by diverse neural circuits. Future improvements in the temporal control, spatial accuracy, and quantitative robustness of these technologies will be crucial for advancing both basic neuroscience and clinical applications. By integrating molecular, cellular, and circuit-level information, PL approaches have the potential to reshape our understanding of the brain and inform the development of targeted therapies for complex brain disorders.

## References

[B1] Bayés À Van De Lagemaat L. N. Collins M. O. Croning M. D. R. Whittle I. R. Choudhary J. S. (2011). Characterization of the proteome, diseases and evolution of the human postsynaptic density. *Nat. Neurosci.* 14 19–21. 10.1038/nn.2719 21170055 PMC3040565

[B2] Boyken J. Grønborg M. Riedel D. Urlaub H. Jahn R. Chua J. J. E. (2013). Molecular profiling of synaptic vesicle docking sites reveals novel proteins but few differences between glutamatergic and GABAergic synapses. *Neuron* 78 285–297. 10.1016/j.neuron.2013.02.027 23622064

[B3] Branon T. C. Bosch J. A. Sanchez A. D. Udeshi N. D. Svinkina T. Carr S. A. (2018). Efficient proximity labeling in living cells and organisms with TurboID. *Nat. Biotechnol.* 36 880–887. 10.1038/nbt.4201 30125270 PMC6126969

[B4] Carlisle H. J. Luong T. N. Medina-Marino A. Schenker L. Khorosheva E. Indersmitten T. (2011). Deletion of Densin-180 results in abnormal behaviors associated with mental illness and reduces mGluR5 and DISC1 in the postsynaptic density fraction. *J. Neurosci.* 31 16194–16207. 10.1523/JNEUROSCI.5877-10.2011 22072671 PMC3235477

[B5] Cho K. F. Branon T. C. Rajeev S. Svinkina T. Udeshi N. D. Thoudam T. (2020). Split-TurboID enables contact-dependent proximity labeling in cells. *Proc. Natl. Acad. Sci.* 117 12143–12154. 10.1073/pnas.1919528117 32424107 PMC7275672

[B6] Cho Y. Jeong I. Kim K. Rhee H.-W. (2025). Painting cell–cell interactions by horseradish peroxidase and endogenously generated hydrogen peroxide. *ACS Chem. Biol.* 20 86–93. 10.1021/acschembio.4c00419 39692451 PMC13459412

[B7] Chong C. H. Li Q. Mak P. H. S. Ng C. C. P. Leung E. H. W. Tan V. H. (2019). Lrrc7 mutant mice model developmental emotional dysregulation that can be alleviated by mGluR5 allosteric modulation. *Transl. Psychiatry* 9:244. 10.1038/s41398-019-0580-9 31582721 PMC6776540

[B8] Cijsouw T. Ramsey A. M. Lam T. T. Carbone B. E. Blanpied T. A. Biederer T. (2018). Mapping the proteome of the synaptic cleft through proximity labeling reveals new cleft proteins. *Proteomes* 6:48. 10.3390/proteomes6040048 30487426 PMC6313906

[B9] De Munter S. Görnemann J. Derua R. Lesage B. Qian J. Heroes E. (2017). Split-BioID: A proximity biotinylation assay for dimerization-dependent protein interactions. *FEBS Lett.* 591 415–424. 10.1002/1873-3468.12548 28032891

[B10] Dieterich D. C. Kreutz M. R. (2016). Proteomics of the synapse – a quantitative approach to neuronal plasticity. *Mol. Cell. Proteomics* 15 368–381. 10.1074/mcp.R115.051482 26307175 PMC4739661

[B11] Falahati H. Wu Y. Feuerer V. Simon H.-G. De Camilli P. (2022). Proximity proteomics of synaptopodin provides insight into the molecular composition of the spine apparatus of dendritic spines. *Proc. Natl. Acad. Sci.* 119:e2203750119. 10.1073/pnas.2203750119 36215465 PMC9586327

[B12] Farizatto K. L. G. Baldwin K. T. (2023). Astrocyte-synapse interactions during brain development. *Curr. Opin. Neurobiol.* 80:102704. 10.1016/j.conb.2023.102704 36913751

[B13] Fernández E. Collins M. O. Uren R. T. Kopanitsa M. V. Komiyama N. H. Croning M. D. R. (2009). Targeted tandem affinity purification of PSD-95 recovers core postsynaptic complexes and schizophrenia susceptibility proteins. *Mol. Syst. Biol.* 5:269. 10.1038/msb.2009.27 19455133 PMC2694677

[B14] Gao Y. Hisey E. Bradshaw T. W. A. Erata E. Brown W. E. Courtland J. L. (2019). Plug-and-play protein modification using homology-independent universal genome engineering. *Neuron* 103 583–597.e8. 10.1016/j.neuron.2019.05.047 31272828 PMC7200071

[B15] Gao Y. Shonai D. Trn M. Zhao J. Soderblom E. J. Garcia-Moreno S. A. (2024). Proximity analysis of native proteomes reveals phenotypic modifiers in a mouse model of autism and related neurodevelopmental conditions. *Nat. Commun.* 15:6801. 10.1038/s41467-024-51037-x 39122707 PMC11316102

[B16] Grant S. G. (2012). Synaptopathies: Diseases of the synaptome. *Curr. Opin. Neurobiol.* 22 522–529. 10.1016/j.conb.2012.02.002 22409856

[B17] Grønborg M. Pavlos N. J. Brunk I. Chua J. J. E. Münster-Wandowski A. Riedel D. (2010). Quantitative comparison of glutamatergic and GABAergic synaptic vesicles unveils selectivity for few proteins including MAL2, a novel synaptic vesicle protein. *J. Neurosci.* 30 2–12. 10.1523/JNEUROSCI.4074-09.2010 20053882 PMC6632534

[B18] Han S. Li J. Ting A. Y. (2018). Proximity labeling: Spatially resolved proteomic mapping for neurobiology. *Curr. Opin. Neurobiol.* 50 17–23. 10.1016/j.conb.2017.10.015 29125959 PMC6726430

[B19] Hindley N. Sanchez Avila A. Henstridge C. (2023). Bringing synapses into focus: Recent advances in synaptic imaging and mass-spectrometry for studying synaptopathy. *Front. Synaptic Neurosci.* 15:1130198. 10.3389/fnsyn.2023.1130198 37008679 PMC10050382

[B20] Irala D. Wang S. Sakers K. Nagendren L. Ulloa Severino F. P. Bindu D. S. (2024). Astrocyte-secreted neurocan controls inhibitory synapse formation and function. *Neuron* 112 1657–1675.e10. 10.1016/j.neuron.2024.03.007 38574730 PMC11098688

[B21] Ito Y. Nagamoto S. Takano T. (2024). Synaptic proteomics decode novel molecular landscape in the brain. *Front. Mol. Neurosci.* 17:1361956. 10.3389/fnmol.2024.1361956 38726307 PMC11079194

[B22] Johnson B. S. Chafin L. Farkas D. Adair J. Elhance A. Farkas L. (2022). MicroID2: A novel biotin ligase enables rapid proximity-dependent proteomics. *Mol. Cell. Proteomics* 21:100256. 10.1016/j.mcpro.2022.100256 35688383 PMC9293651

[B23] Kaizuka T. Suzuki T. Kishi N. Tamada K. Kilimann M. W. Ueyama T. (2024). Remodeling of the postsynaptic proteome in male mice and marmosets during synapse development. *Nat. Commun.* 15:2496. 10.1038/s41467-024-46529-9 38548776 PMC10979008

[B24] Kido K. Yamanaka S. Nakano S. Motani K. Shinohara S. Nozawa A. (2020). AirID, a novel proximity biotinylation enzyme, for analysis of protein–protein interactions. *eLife* 9:e54983. 10.7554/eLife.54983 32391793 PMC7302878

[B25] Kim D. I. Jensen S. C. Noble K. A. Kc B. Roux K. H. Motamedchaboki K. (2016). An improved smaller biotin ligase for BioID proximity labeling. *Mol. Biol. Cell* 27 1188–1196. 10.1091/mbc.E15-12-0844 26912792 PMC4831873

[B26] Koopmans F. Van Nierop P. Andres-Alonso M. Byrnes A. Cijsouw T. Coba M. P. (2019). SynGO: An evidence-based, expert-curated knowledge base for the synapse. *Neuron* 103 217–234.e4. 10.1016/j.neuron.2019.05.002 31171447 PMC6764089

[B27] Kubitz L. Bitsch S. Zhao X. Schmitt K. Deweid L. Roehrig A. (2022). Engineering of ultraID, a compact and hyperactive enzyme for proximity-dependent biotinylation in living cells. *Commun. Biol.* 5:657. 10.1038/s42003-022-03604-5 35788163 PMC9253107

[B28] Lein E. Borm L. E. Linnarsson S. (2017). The promise of spatial transcriptomics for neuroscience in the era of molecular cell typing. *Science* 358 64–69. 10.1126/science.aan6827 28983044

[B29] Lepeta K. Lourenco M. V. Schweitzer B. C. Martino Adami P. V. Banerjee P. Catuara-Solarz S. (2016). Synaptopathies: Synaptic dysfunction in neurological disorders – A review from students to students. *J. Neurochem.* 138 785–805. 10.1111/jnc.13713 27333343 PMC5095804

[B30] Loh K. H. Stawski P. S. Draycott A. S. Udeshi N. D. Lehrman E. K. Wilton D. K. (2016). Proteomic analysis of unbounded cellular compartments: Synaptic clefts. *Cell* 166 1295–1307.e21. 10.1016/j.cell.2016.07.041 27565350 PMC5167540

[B31] Martell J. D. Yamagata M. Deerinck T. J. Phan S. Kwa C. G. Ellisman M. H. (2016). A split horseradish peroxidase for the detection of intercellular protein–protein interactions and sensitive visualization of synapses. *Nat. Biotechnol.* 34 774–780. 10.1038/nbt.3563 27240195 PMC4942342

[B32] Morciano M. Beckhaus T. Karas M. Zimmermann H. Volknandt W. (2009). The proteome of the presynaptic active zone: From docked synaptic vesicles to adhesion molecules and maxi-channels. *J. Neurochem.* 108 662–675. 10.1111/j.1471-4159.2008.05824.x 19187093

[B33] Newman Z. L. Bakshinskaya D. Schultz R. Kenny S. J. Moon S. Aghi K. (2022). Determinants of synapse diversity revealed by super-resolution quantal transmission and active zone imaging. *Nat. Commun.* 13:229. 10.1038/s41467-021-27815-2 35017509 PMC8752601

[B34] O’Rourke N. A. Weiler N. C. Micheva K. D. Smith S. J. (2012). Deep molecular diversity of mammalian synapses: Why it matters and how to measure it. *Nat. Rev. Neurosci.* 13 365–379. 10.1038/nrn3170 22573027 PMC3670986

[B35] Pascual-Caro C. De Juan-Sanz J. (2024). Monitoring of activity-driven trafficking of endogenous synaptic proteins through proximity labeling. *PLoS Biol.* 22:e3002860. 10.1371/journal.pbio.3002860 39466808 PMC11542813

[B36] Raghunathan K. Eroglu C. (2025). Developmental roles of astrocytes in circuit wiring. *Curr. Opin. Neurobiol.* 92:103042. 10.1016/j.conb.2025.103042 40367704 PMC12162199

[B37] Ramanathan M. Majzoub K. Rao D. S. Neela P. H. Zarnegar B. J. Mondal S. (2018). RNA–protein interaction detection in living cells. *Nat. Methods* 15 207–212. 10.1038/nmeth.4601 29400715 PMC5886736

[B38] Rosenthal J. S. Zhang D. Yin J. Long C. Yang G. Li Y. (2025). Molecular organization of central cholinergic synapses. *Proc. Natl. Acad. Sci.* 122:e2422173122. 10.1073/pnas.2422173122 40273107 PMC12054790

[B39] Schopp I. M. Amaya Ramirez C. C. Debeljak J. Kreibich E. Skribbe M. Wild K. (2017). Split-BioID a conditional proteomics approach to monitor the composition of spatiotemporally defined protein complexes. *Nat. Commun.* 8:15690. 10.1038/ncomms15690 28585547 PMC5467174

[B40] Spence E. F. Dube S. Uezu A. Locke M. Soderblom E. J. Soderling S. H. (2019). In vivo proximity proteomics of nascent synapses reveals a novel regulator of cytoskeleton-mediated synaptic maturation. *Nat. Commun.* 10:386. 10.1038/s41467-019-08288-w 30674877 PMC6344529

[B41] Strack S. Robison A. J. Bass M. A. Colbran R. J. (2000). Association of calcium/calmodulin-dependent kinase II with developmentally regulated splice variants of the postsynaptic density protein densin-180. *J. Biol. Chem.* 275 25061–25064. 10.1074/jbc.C000319200 10827168

[B42] Takano T. Soderling S. H. (2021). Tripartite synaptomics: Cell-surface proximity labeling in vivo. *Neurosci. Res.* 173 14–21. 10.1016/j.neures.2021.05.002 34019951 PMC8602446

[B43] Takano T. Wallace J. T. Baldwin K. T. Purkey A. M. Uezu A. Courtland J. L. (2020). Chemico-genetic discovery of astrocytic control of inhibition in vivo. *Nature* 588 296–302. 10.1038/s41586-020-2926-0 33177716 PMC8011649

[B44] Tetenborg S. Shihabeddin E. Kumar E. O. A. M. Sigulinsky C. L. Dedek K. Lin Y. P. (2025). Uncovering the electrical synapse proteome in retinal neurons via in vivo proximity labeling. *BioRxiv[Preprint]* 10.1101/2024.11.26.625481 39651118 PMC11623651

[B45] Uezu A. Kanak D. J. Bradshaw T. W. A. Soderblom E. J. Catavero C. M. Burette A. C. (2016). Identification of an elaborate complex mediating postsynaptic inhibition. *Science* 353 1123–1129. 10.1126/science.aag0821 27609886 PMC5432043

[B46] Unterauer E. M. Shetab Boushehri S. Jevdokimenko K. Masullo L. A. Ganji M. Sograte-Idrissi S. (2024). Spatial proteomics in neurons at single-protein resolution. *Cell* 187 1785–1800.e16. 10.1016/j.cell.2024.02.045 38552614

[B47] Van DeusenA. L.KumarS.CalhanO. Y.GogginS. M.ShiJ.WilliamsC. M. (2025). A single-cell mass cytometry-based atlas of the developing mouse brain. *Nat. Neurosci.* 28 174–188. 10.1038/s41593-024-01786-1 39695302

[B48] Van Oostrum M. Blok T. M. Giandomenico S. L. Tom Dieck S. Tushev G. Fürst N. (2023). The proteomic landscape of synaptic diversity across brain regions and cell types. *Cell* 186 5411–5427.e23. 10.1016/j.cell.2023.09.028 37918396 PMC10686415

[B49] Van Oostrum M. Schuman E. M. (2025). Understanding the molecular diversity of synapses. *Nat. Rev. Neurosci.* 26 65–81. 10.1038/s41583-024-00888-w 39638892

[B50] Wang S. Baumert R. Séjourné G. Sivadasan Bindu D. Dimond K. Sakers K. (2023). PD-linked LRRK2 G2019S mutation impairs astrocyte morphology and synapse maintenance via ERM hyperphosphorylation. *BioRxiv [Preprint]* 10.1101/2023.04.09.536178 39253496 PMC11383028

[B51] Wilhelm B. G. Mandad S. Truckenbrodt S. Kröhnert K. Schäfer C. Rammner B. (2014). Composition of isolated synaptic boutons reveals the amounts of vesicle trafficking proteins. *Science* 344 1023–1028. 10.1126/science.1252884 24876496

[B52] Willim J. Woike D. Greene D. Das S. Pfeifer K. Yuan W. (2024). Variants in LRRC7 lead to intellectual disability, autism, aggression and abnormal eating behaviors. *Nat. Commun.* 15:7909. 10.1038/s41467-024-52095-x 39256359 PMC11387733

[B53] Xu Y. Song X. Wang D. Wang Y. Li P. Li J. (2021). Proteomic insights into synaptic signaling in the brain: The past, present and future. *Mol. Brain* 14:37. 10.1186/s13041-021-00750-5 33596935 PMC7888154

[B54] Yao Z. Van Velthoven C. T. J. Kunst M. Zhang M. McMillen D. Lee C. (2023). A high-resolution transcriptomic and spatial atlas of cell types in the whole mouse brain. *Nature* 624 317–332. 10.1038/s41586-023-06812-z 38092916 PMC10719114

[B55] Yuan C. Patel K. Shi H. Wang H.-L. V. Wang F. Li R. (2025). mcDETECT: Decoding 3D spatial synaptic transcriptomes with subcellular-resolution spatial transcriptomics. *BioRxiv[Preprint*] 10.1101/2025.03.27.645744 40236251 PMC11996425

[B56] Zhang M. Pan X. Jung W. Halpern A. R. Eichhorn S. W. Lei Z. (2023). Molecularly defined and spatially resolved cell atlas of the whole mouse brain. *Nature* 624 343–354. 10.1038/s41586-023-06808-9 38092912 PMC10719103

[B57] Zhang Z. Wang Y. Lu W. Wang X. Guo H. Pan X. (2025). Spatiotemporally resolved mapping of extracellular proteomes via in vivo-compatible TyroID. *Nat. Commun.* 16:2553. 10.1038/s41467-025-57767-w 40089463 PMC11910615

